# Anti-cachectic effect of *Antrodia cinnamomea* extract in lung tumor-bearing mice under chemotherapy

**DOI:** 10.18632/oncotarget.24680

**Published:** 2018-04-13

**Authors:** Meng-Chuan Chen, Wen-Lin Hsu, Tz-Chong Chou

**Affiliations:** ^1^ School of Dentistry, Graduated Institute of Dental Science, National Defense Medical Center, Taipei, Taiwan; ^2^ Department of Radiation Oncology, Buddhist Tzu Chi General Hospital, Hualien, Taiwan; ^3^ School of Medicine, Tzu Chi University, Hualien, Taiwan; ^4^ Cancer Research Center, Buddhist Tzu Chi General Hospital, Hualien, Taiwan; ^5^ Institute of Medical Sciences, Tzu Chi University, Hualien, Taiwan; ^6^ Department of Biotechnology, Asia University, Taichung, Taiwan; ^7^ China Medical University Hospital, China Medical University, Taichung, Taiwan

**Keywords:** *Antrodia cinnamomea*, lung tumor, cancer cachexia, muscle atrophy, chemotherapy

## Abstract

Skeletal muscle atrophy, the most characteristic feature of cancer cachexia, often occurs in patients with cancer undergoing chemotherapy. *Antrodia cinnamomea* (AC) a widely used edible medical fungus, exhibits hepatoprotective, anti-inflammatory and anticancer activities. In this study, we investigated whether combined treatment with the ethonolic extract of AC ameliorates cachexia symptoms, especially muscle wasting, in lung tumor-bearing mice treated with chemotherapy. Our results revealed that gemcitabine and cisplatin-induced severe body weight loss and skeletal muscle atrophy in the mice with cancer were greatly attenuated after AC extract administration. The protection may be attributed to the inhibition of skeletal muscle proteolysis by suppressing myostatin and activin release, muscle wasting-related FoxO3/MuRF-1/MAFbx signaling, proteasomal enzyme activity, and pro-inflammatory cytokine production. A significant decrease in insulin-like growth factor 1 (IGF-1) expression and formation was observed in the atrophying muscle of the conventional chemotherapy treatment group (CGC), and this decrease was markedly reversed by AC treatment. Additionally, the anorexia, intestinal injury and dysfunction that occurred in the CGC group were mitigated by AC extract. Taken together, these results demonstrated that the AC extract has a protective effect against chemotherapy-induced muscle atrophy mainly by attenuating muscle proteolysis, pro-inflammatory cytokine production, and anorexia, and activating IGF-1-dependent protein synthesis.

## INTRODUCTION

Cancer cachexia is characterized by body weight loss, anorexia, fatigue, inflammation and abnormal metabolism, thereby markedly reducing quality of life and limiting the application of conventional therapy such as chemotherapy [1, 2]. More than 80% of patients with advanced cancer suffer from cachexia, and cancer cachexia is estimated to account for at least 20% of deaths in patients with cancer [3, 4]. Thus, how to prevent and attenuate the development of cancer cachexia is a key concern during cancer therapy. Muscle wasting, the most prominent phenotypic feature of cancer cachexia, is closely related to the tumor site, size, stage, and treatment type [5]. During cachexia, muscle proteolysis is predominately triggered by the ubiquitin proteasome system (UPS). The forkhead box O (FoxO) transcription factor is a key factor for activating the expression of muscle-specific ubiquitin conjugating enzymes E3 ligase, F-box (MAFbx)/atrogin-1 and muscle ring finger 1 (MuRF-1). The ubiquitinated target protein substrate can be recognized by the 26S proteasome and then digested to peptides, which in turn leads to muscle protein degradation [6]. Increased ubiquitinated protein expression and proteasome activity have been observed in atrophying muscle [7]. Conversely, mice that are deficient in either MAFbx or MuRF-1 exhibit greater resistance to atrophy [8], suggesting that suppressing the FoxO/MAFbx/MuRF-1/UPS cascade is a promising strategy of preventing the muscle wasting associated with cancer cachexia. The process of cancer cachexia-evoked muscle atrophy is complex and multifactorial; it is mediated by the interplay of tumor factors, host factors, and the interaction between the two [9, 10].

To date, chemotherapy remains a widely used option for cancer therapy. Nevertheless, several deleterious effects have been identified after chemotherapy, thereby limiting its application [11]. Currently, combined treatment with chemotherapeutic drugs such as gemcitabine (2’, 2’-difluorodeoxycytidine) and cisplatin (cis-diammine-dichloroplatinum) is a common regimen for treating metastatic lung cancer. However, many side effects, including nephrotoxicity, gastrointestinal mucosal injury, and severe muscle wasting, have been reported in patients with cancer treated with cisplatin [12, 13]. These findings imply that chemotherapy itself can induce muscle atrophy [14]. Although various drugs and supplements have been used, cancer cachexia-evoked skeletal muscle mass loss remains a major problem. Therefore, developing more effective and safe chemotherapeutic adjuvants or nutritional supplements to ameliorate chemotherapy-induced muscle wasting is urgent.

*Antrodia cinnamomea* (AC), a medical fungus, grows only on the inner cavity of the endemic species *Cinnamomum kanehirai,* which is a plant native to Taiwan. AC extracts have been demonatrated to possess several beneficial effects, including antioxidant, hepato-protective, anti-hypertensive, anti-hyperlipidemic, immunomodulatory, anticancer, and anti-inflammatory activities [15, 16]. However, the effects of AC extract on cancer cachexia remain unknown. Therefore, the aim of this study was to examine whether AC attenuates these cachectic symptoms, particularly the muscle atrophy, in lung tumor-bearing mice under chemotherapy, and to further investigate the molecular mechanisms involved.

## RESULTS

### Chemical characteristics of the AC extract and its effects on tumor growth

**Figure 1:**
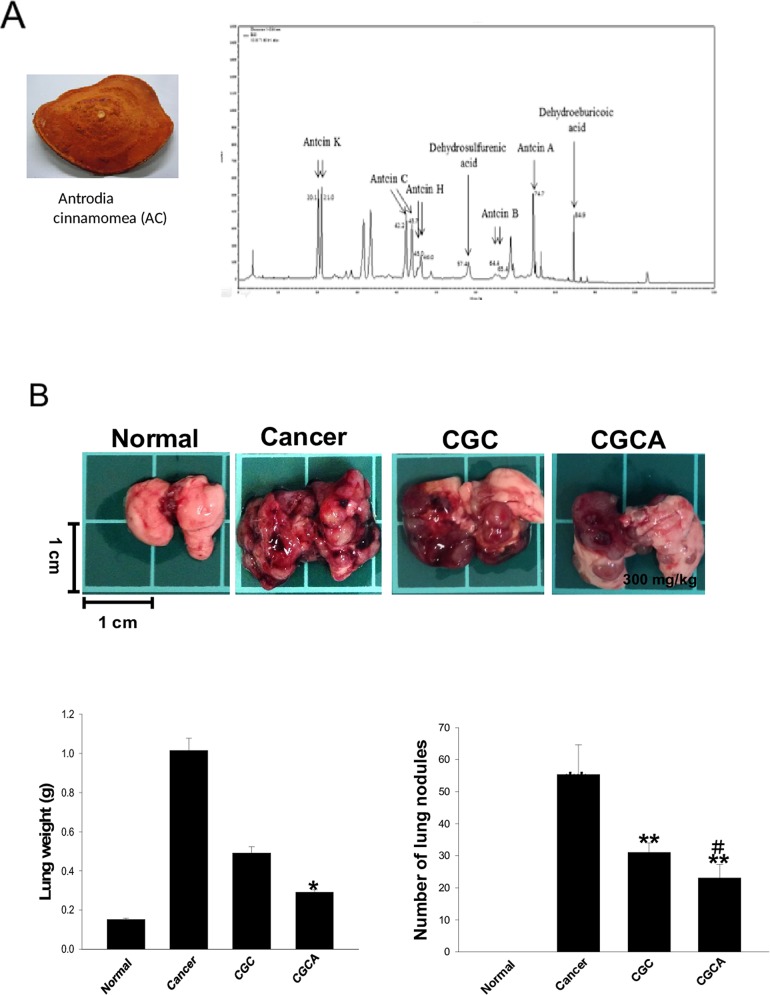
The chemical components of the AC extract and its effects on lung tumor growth The morphology of the fruiting bodies of *Antrodia cinnamomea* (AC) and the representative HPLC profile of the ethanolic extract of AC **(A)**. The images of tumors, the weight, and the tumor nodules of lungs were measured in various groups **(B)**. Data was expressed as mean ± S.E.M. (n=5). ^*^P < 0.05, ^**^P < 0.01, ^***^P < 0.001 versus the normal group. ^#^*P* < 0.05 versus the CGC group.

Data from the HPLC analysis revealed that the ethanolic extract of the patented kinetic bio-activation fruiting body of AC contained seven marker triterpenoid ingredients, namely antcin A (at 74.7 min), antcin B (at 64.4 and 65.4 min), antcin C (at 42.2 and 45.7 min), antcin H (at 45.0 min), antcin K (at 20.1 and 21.0 min), dehydrosulphurenic acid (at 57.4 min) and dehydroeburicoic acid (at 84.9 min) (Figure 1A). The total amount of the triterpenoids was observed to account for 14.5% (w/w) of the AC extract, and antcin C, antcin H, and antcin K were abundant among these triterpenoids (Table 1). Four groups were used in this study: (1) a normal group; (2) cancer group (tumor-alone group); (3) CGC group: mice treated with a standard diet and an intraperitoneal injection of gemcitabine (1000 mg/m^2^ per 3 days) and cisplatin (75 mg/m^2^/week); (4) CGCA group: mice treated with a standard diet plus AC extract (300 mg/kg/day, p.o.) and an intraperitoneal injection of gemcitabine and cisplatin. After orthotopic implantation of LLC2 cells into the lungs for two weeks, the mice were treated with different combinations of drugs for 21 days and were sacrificed to evaluate tumor progression. The number of tumor nodules and the lung weight reflecting the tumor growth were greatly reduced in the CGC and CGCA groups, compared with the cancer alone group. Furthermore, we observed that the anticancer activity of the CGCA group was stronger than that of the CGC group (Figure 1B).

**Table 1:** The contents of triterpenoid compounds in the ethanol extract of AC determined by LC-MS/MS method

Compound name	LC/MS quantitative result, ppm
Antcin A	5,196
Antcin B	3,363
Antcin C	58,274
Antcin H	15,565
Antcin K	52,679
dehydrosulphurenic acid	5,765
dehydroeburicoic acid	3,699
Total	144,541

### AC attenuates muscle atrophy and proteasome activity in muscle

**Figure 2:**
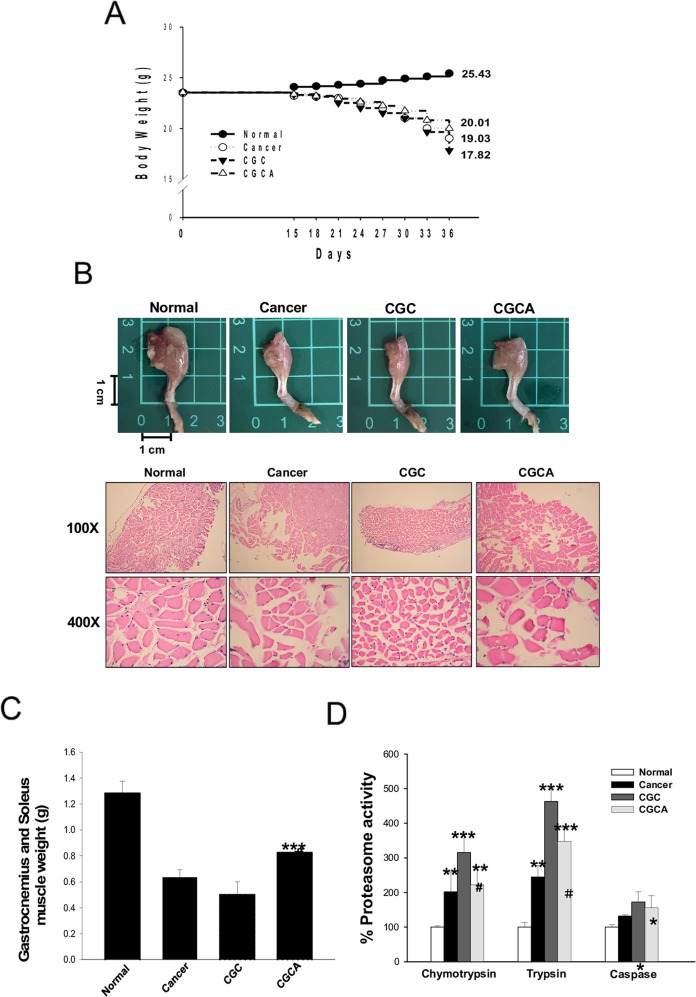
AC treatment attenuates body weight loss and muscle atrophy The body weight **(A)**, a representative image of the muscle of limb **(B)**, the weight of gastrocnemius and soleus muscle **(C)**, and the proteasome activity **(D)** were photographed or measured in various groups. Data was expressed as mean ± S.E.M. (n=5). ^*^P < 0.05, ^**^P < 0.01, ^***^P < 0.001 versus the normal group.^#^*P* < 0.05, ^##^*P* < 0.01 versus the CGC group.

At the end of the study, the untreated cancer mice lost 19.0 ± 1.4% of their initial body weight, whereas the normal mice gained body weight. The mice in the CGC and CGCA groups lost 24.2 ± 1.6%, and 18.8 ± 1.8% of their initial weight, respectively (Figure 2A). During cancer cachexia, body weight loss generally results from skeletal muscle wasting. As expected, the muscle atrophy evaluated through histological examination was parallel with the trend of muscle mass loss in these groups (Figure 2B). Notably, the CGC group had the most skeletal muscle mass loss, as evidenced by a marked reduction in the weight of the gastrocnemius and soleus muscle, and this loss was inhibited by combined treatment with AC (CGCA) (Figure 2C). Consistently, a marked increase in muscular proteasome activity, particularly chymotrypsin and trypsin, was observed in the CGC group, and this increase was significantly inhibited by AC treatment (Figure 2D).

### AC inhibits muscle wasting-related signaling pathway

**Figure 3:**
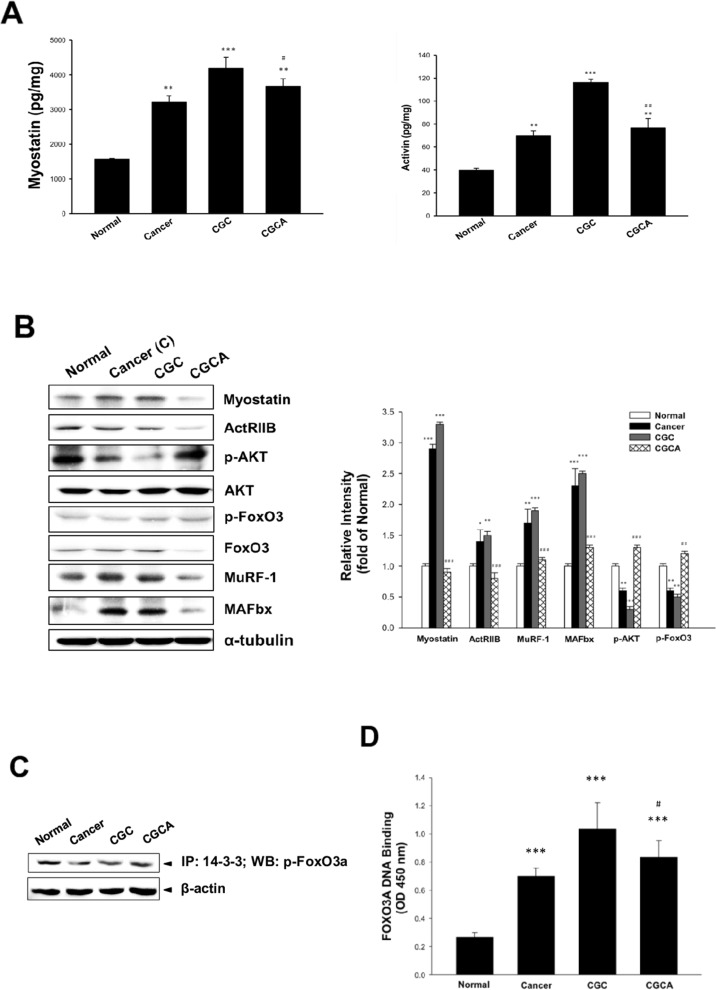
Effects of AC on muscle atrophy-related mediator formation and gene expression The formation of myostatin and activin A in muscle **(A)** and various atrogenic gene expression **(B)** were determined. The association of p-FoxO3a with 14-3-3 chaperone protein **(C)** and FoxO3a transcription factor activity **(D)** in muscle were examined. Data was expressed as mean ±S.E.M. (n=5). ^*^P < 0.05, ^**^P < 0.01, ^***^P < 0.001 versus the normal group. ^#^*P* < 0.05, ^##^*P* < 0.01 versus the CGC group.

Overproduction of myostatin and activin A observed in the atrophying muscle of the cancer-alone and CGC groups was significantly decreased in the mice in the CGCA group (Figure 3A). Similarly, the alterations in muscle wasting-related gene expression, including increased levels of ActRIIB, FoxO3, MuRF 1, and MAFbx, as well as decreased expression of p-Akt and p-FoxO3, particularly in the muscle of the CGC group, were markedly reversed through AC treatment (Figure 3B). The 14-3-3 chaperone protein binds to phosphorylated FoxO3, resulting in FoxO3 degradation, thereby inhibiting downstream MuRF 1 and MAFbx expression [17]. Our results confirmed that the interaction of p-FoxO3 with the 14-3-3 protein determined by an immunoprecipitation assay was increased in the CGCA group, compared with the cancer alone and CGC groups (Figure 3C). As expected, a marked elevation of FoxO3 transcriptional activity in the CGC group was significantly inhibited by AC treatment (Figure 3D). Therefore, suppressing FoxO3-mediated processes may contribute to the attenuation of muscle proteolysis by AC.

### AC inhibits pro-inflammatory cytokine production and upregulates insulin-like growth factor-1 (IGF-1)

**Figure 4:**
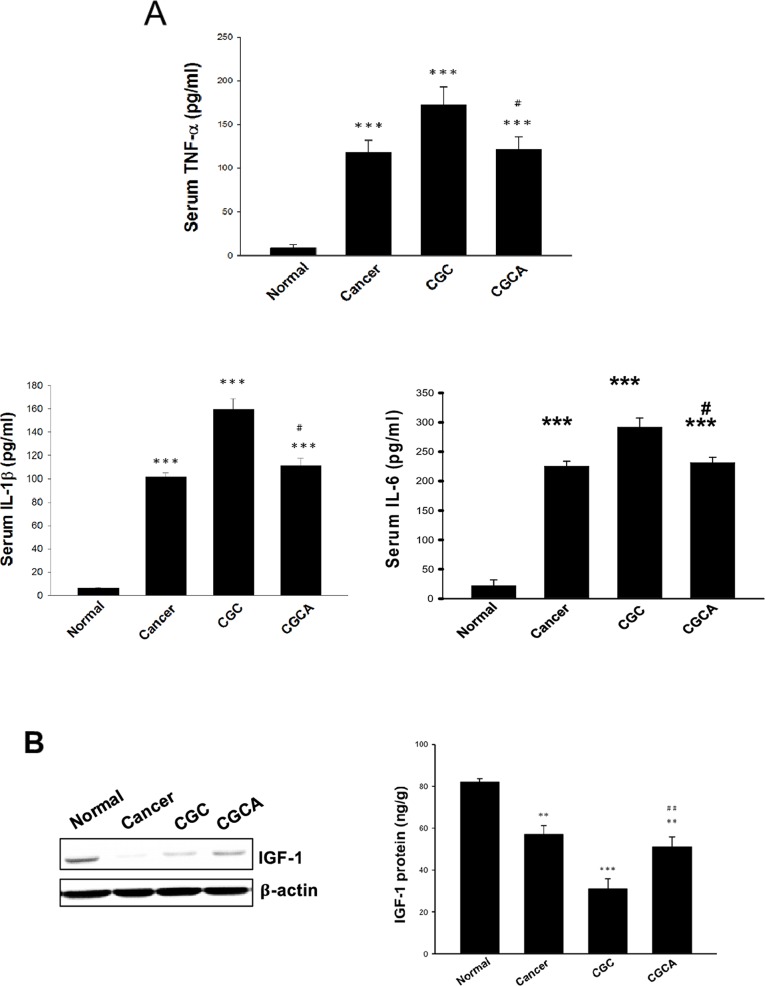
Effects of AC on serum pro-inflammatory cytokine levels and IGF-1 expression The serum levels of pro-inflammatory cytokines **(A)**, and the expression and amount of IGF-1 in muscle of different groups were measured **(B)**. Data was expressed as mean ±S.E.M. (n=5). ^**^P < 0.01, ^***^P < 0.001 versus the normal group. ^#^*P* < 0.05, ^##^*P* < 0.01 versus the CGC group.

Systemic inflammation is considered a key factor inducing cancer cachexia [18]. A significant elevation of serum levels of pro-inflammatory cytokines, including TNF-α, IL-6 and IL-1β, particularly in the CGC group, was greatly diminished in the CGCA group (Figure 4A). Notably, combined treatment with the AC extract greatly increased the expression and production of IGF-1, compared with the CGC group (Figure 4B). These results suggest that inhibiting inflammatory responses and activating IGF-1-dependent process involves the antiatrophic effect of AC.

### AC prevents intestinal injury/dysfunction

**Figure 5:**
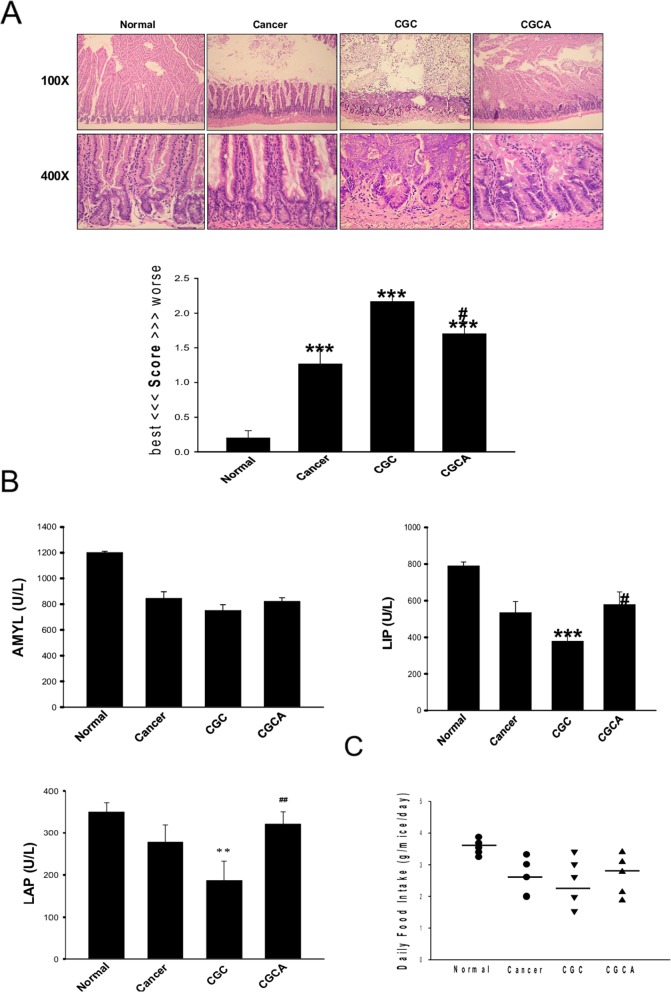
Treatment with AC ameliorates intestinal damage and digestive enzyme dysfunction The morphological changes and the grading score of the damage in intestines of different groups were evaluated **(A)**. Various intestinal digestive enzyme activity **(B)** and the daily food intake were measured **(C)**. Data was expressed as mean ±S.E.M. (n=5). ^**^P < 0.01, ^***^P < 0.001 versus the normal group. ^#^*P* < 0.05, ^##^*P* < 0.01 versus the CGC group.

Severe damage to the intestinal mucosal structure, especially in the CGC group, was ameliorated in the mice in the CGCA group (Figure 5A). Additionally, impaired digestive enzyme activity, such as leucine amino peptidase (LAP), a digestive enzyme for peptides, amylase (AMYL), a digestive enzyme for sugars, and lipase (LIP), a digestive enzyme for fat, in the cancer-alone and CGC groups, was alleviated after AC administration (Figure 5B). Notably, AC could mitigate the anorexia observed in the CGC group, as evidenced by an elevation in daily food intake (Figure 5C).

## DISCUSSION

Several tumor and host factors play critical roles in triggering muscle atrophy associated with cancer cachexia by activating muscle proteolytic pathways, impairing protein synthesis, or both [19, 20]. The current study demonstrates, for the first time, that combined treatment with the ethanolic extract of AC greatly alleviates gemcitabine and cisplatin-induced cachectic symptoms, including body weight loss, skeletal muscle atrophy, intestinal damage and dysfunction, and anorexia in lung tumor-bearing mice; this thus supports the clinical use of this extract. Myostatin belonging to transforming growth factor-α; (TGF-α) superfamily is mostly expressed in skeletal muscle, and it can inhibit skeletal muscle growth by reducing myoblast proliferation and myogenesis [21]. Conversely, blocking the actions of myostatin by using neutralizing antibodies or antagonists remarkably increases muscle size and physical strength [22]. Similarly, activins, members of the TGF-α superfamily, are another muscle atrophying factor. Notably, myostatin and activins can bind the same muscle surface receptor complex that composes of type-II activin receptors (ActRIIA and ActRIIB) and type-I activin receptors (ALK4 and ALK5), which ultimately results in muscle proteolysis through activation of FoxO, especially the FoxO3 isoform. The actions of FoxO are largely controlled by the subcellular localization and the protein degradation of FoxO. When FoxO is phosphorylated and inactivated by Akt, the phosphorylated FoxO is exported from the nucleus into cytoplasma in a chaperone 14-3-3-dependent manner [17]. The 14-3-3 bound phosphorylated FoxO protein is then degraded through proteasome. In response to myostatin and activin, Akt activity is inhibited, leading to the accumulation of the dephospho-FoxO protein, the active form of FoxO. Subsequently, the activated FoxO translocates into the nucleus and enhances the transcription of muscle-specific atrogenic genes, such as MuRF-1 and MAFbx. Previous studies have reported that myostatin and activin signaling in muscle increases in patients with cancer suffering from cachexia and in experimental cancer cachexia [23, 24]. In the present study, we demonstrated that the levels of muscle myostatin and activin were significantly reduced in the CGCA group, compared with those in the CGC group. An increase in ActRIIB expression and a decrease in Akt phosphorylation in the CGC group were also reversed by AC treatment, which may be associated with the inhibition of myostatin and activin generation. As expected, treatment with AC increased FoxO3 phosphorylation accompanied by an elevation of the association of 14-3-3 with phospho-FoxO3 in cytoplasma, but it significantly inhibited MuRF-1 and MAFbx expression, and proteasome activity. Collectively, suppressing the myostatin/activins/FoxO3/MuRF-1/MAFbx signaling pathway may contribute to the antiatrophic effect of AC.

Accumulating evidence indicates that systemic inflammation and pro-inflammatory cytokines, including TNF-β, IL-6 and IL-1β, are major factors in promoting muscle atrophy through UPS activation, muscle differentiation and myogenesis inhibition [18], and improvement of anorexia during cancer cachexia [25, 26]. Elevated serum levels of pro-inflammatory cytokines were reported in patients with cachexia [27]. However, neutralization of pro-inflammatory cytokines could markedly relieve the cachectic symptoms in an experimental animal model [28]. According to the results that the CGCA group had lower serum levels of NF-β, IL-6, and IL-1β than that in the CGC group, inhibition of pro-inflammatory cytokine formation may also involve the anti-cachectic effects of AC.

In addition to muscle proteolysis, muscle protein generation is another crucial factor in regulating muscle mass. A central role of IGF-1 in enhancing muscle growth by activating PI3K/Akt/mTOR-regulated protein synthesis was demonstrated, previously [29]. Moreover, IGF-1 can activate Akt-induced FoxO phosphorylation, thereby attenuating muscle protein degradation [30]. Therefore, IGF-1 not only enhances protein synthesis but also inhibits muscle proteolysis, suggesting that IGF-1induction is a promising approach to prevent muscle atrophy. A novel finding of this study is that the CGCA group had a higher IGF-1 level in muscle than that of the CGC group. Accordingly, AC-mediated attenuation of muscle mass loss may, at least partly, be attributed to the suppression of inflammation-evoked muscle protein degradation, and enhancement of IGF-1-dependent protein synthesis.

Maintaining the intestinal structure and function is essential for nutritional intake and body growth. Notably, intestinal damage and impaired digestive enzyme activity, particularly in the CGC group, were greatly improved through AC treatment. Thus, increased nutrient absorption and availability due to the attenuation of intestinal injury and dysfunction may in turn enhance appetite, as reflected by increased daily food intake, thereby attenuating body weight loss by AC in this cachectic model. Notably, the inhibition of lung tumor growth through combined treatment with AC was stronger than that observed in the CGC group, suggesting that AC can also enhance the anticancer activity of chemotherapy.

Ergostane-type triterpenoids are the major bioactive components in the fruiting bodies of AC. Among these triterpenoids, antcin C, antcin H, antcin K, and antcin A have been reported to exhibit anti-inflammatory and anticancer activities [16, 31, 32]. The pharmacokinetic and metabolism study demonstrated that several triterpenoids and metabolites were detected in the plasma of rats after oral administration of the ethanol extract of AC (1g/kg). Generally, the ergostane triterpenoids are absorbed and eliminated rapidly, and the pharmacokinetic patterns of Antrodia triterpenoids are closely related to their chemical structure. The high-polarity of antcin H and antcin K were determined to be metabolically stable, and had markedly higher plasma concentrations than antcin B and antcin C [33]. Conversely, antcin A had a low concentration in plasma. Composition analysis revealed that antcin H and antcin K were abundant in the AC extract. Therefore, antcin K and antcin H may be most responsible for the therapeutic effects on cancer cachexia because of their pharmacokinetic parameters, abundance, and biological effects. However, more basic and human studies are required to determine their clinical use. Notably, the hydrogenated metabolites derived from antcin C also revealed a high plasma concentration. However, the biological functions are still unknown, and need further investigation.

**Figure 6:**
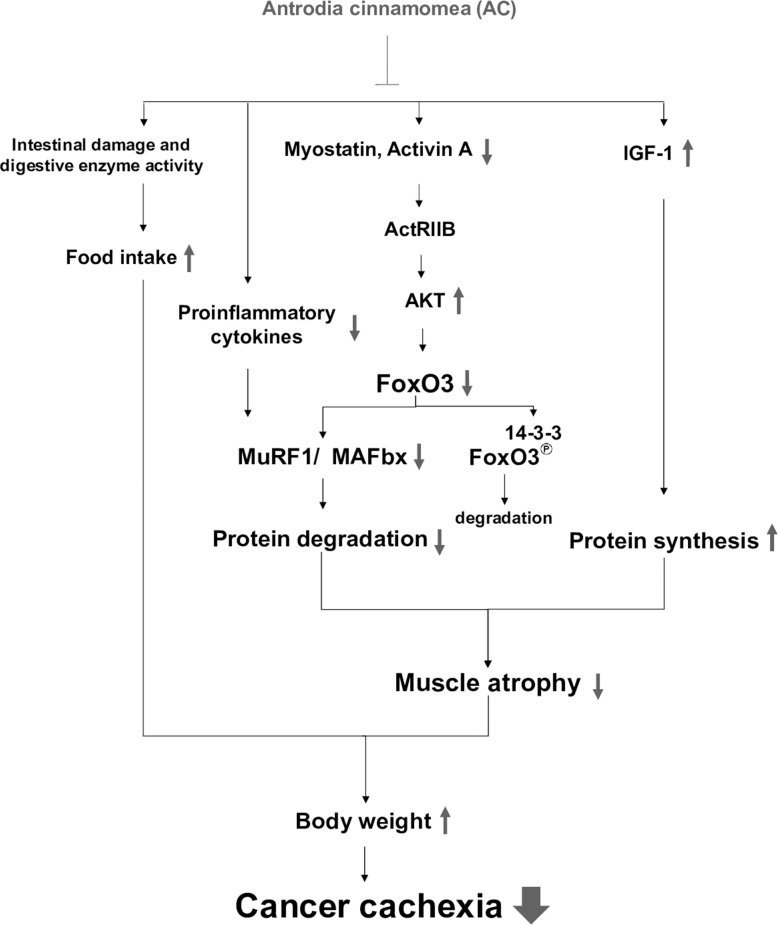
The proposed mechanisms accounting for the anti-cachectic activity of the AC extract Combined treatment with AC inhibits myostatin/activin/FoxO3 signaling and pro-inflammatory cytokine formation, leading to the suppression of MAFbx and MuRF1 expression and proteasome activity in muscle, which in turn attenuates muscle proteolysis. Meanwhile, the AC extract enhances IGF-1 expression and its regulated protein synthesis, and improves anorexia and intestinal damage and dysfunction. Therefore, AC has potential to ameliorate cachectic symptoms under chemotherapy, especially body weight loss.

In summary, AC administration can attenuate the development of cancer cachexia in lung tumor-bearing mice undergoing chemotherapy. Mechanically, the protective effects of AC against muscle wasting may be associated with the suppression of muscle proteolysis, formation of pro-inflammatory cytokines, activation of IGF-1-regulated protein synthesis, and improvement of intestinal damage and dysfunction (Figure 6). Overall, AC has potential to ameliorate cachectic symptoms in patients undergoing chemotherapy.

## MATERIALS AND METHODS

### Preparation of AC extract and reagents

The fruiting bodies of AC were provided by Balay Biotechnology, Inc. (Taipei, Taiwan) and identified by the Bioresource Collection and Research Center (BCRC, Taiwan). The dried AC powder (30g) was soaked with 0.9 L of distilled water at 80°C-100°C for 1 h. Subsequently, it was extracted with 900 mL of ethanol (95%) for 24 h. The residue was filtered through a Buchner funnel lined with Whatman filter paper; the filtrates were collected and dried using a rotary evaporator (CES-800, Panchum Scientific Corp.) under reduced pressure at 45°C to obtain brownish colored residues with a yield of 7.5 g (25%, w/w) and were stored at -20°C prior to analysis. Antibodies, including anti-TNF-α, anti-IL-1β, anti-IGF-1, anti-MuRF-1, anti-MAFbX-1, and anti-β-actin were purchased from Santa Cruz Biotechnology (CA, USA). Anti-Akt, anti-phospho-Akt, anti-phospho-FoxO3a, and anti-FoxO3a were purchased from Cell Signaling Technology (Danvers, MA, USA). Anti-myostatin and anti-IL-6 were purchased from GeneTex, Inc. (CA, USA). Horseradish peroxidase (HRP)-labeled secondary antibody was obtained from Abcam (Cambridge, MA, USA). Cisplatin and gemcitabine were provided by Eli Lilly (Indianapolis, IN, USA). The enzyme-linked immunosorbent assay (ELISA) kits of myostatin, activin A, IGF-1, TNF-α, IL-6, and IL-1β were purchased from R&D Systems, Inc. (MN, USA). Other reagents were purchased from Sigma-Aldrich Corporation (St. Louis, MO, USA).

### Cell culture

The Lewis lung carcinoma cell line LLC2 purchased from the Bioresource Collection and Research Center (Taipei, Taiwan) was incubated in Dulbecco's modified Eagle's Medium (Gibco, Carlsbad, CA, USA) supplemented with 10 % fetal bovine serum (Thermo Fisher Scientific Inc), 2 mmol/L L-glutamine, and 100 U/mL penicillin-streptomycin (Gibco, Carlsbad, CA, USA). Cells were maintained in an incubator with room air: CO_2_ (95:5, v/v) at 37°C.

### Animal model

Seven-week-old male C57B/6 mice weighing approximately 25 g were used for the study. The animal care and experimental procedures were conducted in accordance with the Guiding Principle in the Care and Use of Animals and approved by the Institutional Animal Care and Use Committee of National Defense Medical Center (IACUC 12156). The mice were anaesthetized with an intraperitoneal injection of ketamine HCl/xylazine (100 mg/15 mg body weight per mouse). Anesthetized mice were placed on a platform by their front teeth so that their chests hung vertically beneath them. Light was directed on each mouse's upper chest, on a spot marked by an “X”. The mouth was opened using the Exel Safelet IV catheter, and the tongue is gently pulled out using flat forceps. After locating the white light emitted from the trachea, the Exel Safelet IV catheter was slid into the trachea, and the needle was removed. The mouse with the inserted catheter on the platform is moved into a biosafety hood, where the LLC2 cell (1 × 10^6^ cells/100 μL) was dispensed into the opening of the catheter [34]. After implantation of cancer cells for 10 days, the mice were divided into four weight-matched groups: (1) the normal group, (2) cancer-alone group, (3) CGC group, and (4) CGCA, as described in the Results section. Each group contains five mice.

### Western blotting

Protein samples (100 μg) were separated on a 10% SDS-PAGE, and transferred onto nitrocellulose membranes. After blocking with 5% nonfat dry milk in 5% Tris-buffered saline with Tween 20 (TBST) for 1 h, the membranes were incubated with various appropriately diluted primary antibodies for target genes at 4°C overnight. After washing with TBST, the membranes were incubated with horseradish peroxidase-conjugated secondary antibody for 1 h, and immunoreactivity was visualized using enhanced HRP substrate luminol reagent (Milipore, Billerica, MA, USA).

### Proteasome activity assay

Skeletal muscle (gastrocnemius muscle) samples of approximately 5 mg were dissected from mice, and rinsed in ice-cold phosphate-buffered saline to remove blood. A proteasome activity assay for chymotrypsin, trypsin and caspase was performed using a commercially available Proteasome-Glo™ 3-Substrate Systems kit in accordance with the manufacturer's instruction.

### Co-immunoprecipitation (Co-IP) assay

Sample lysates (1 mg) of the lung were incubated with anti-14-3-3 antibody in 300 μL of ice-cold lysis buffer containing 50 mM Tris–Cl (pH = 7.5), 150 mM NaCl, 1% Nonidet P-40, and 10% glycerol, and freshly supplemented with a protease inhibitor cocktail (Thermo Fisher Scientific Inc.) containing 1 mM DTT, 1 mM EDTA, and 1 mM PMSF. After rocking for 24 h at 4°C, 60 ml of Protein A magnetic beads (Millipore Corporation, Billerica, MA, USA) was added. The mixtures were incubated overnight at 4°C and washed four times with lysis buffer. The precipitates were boiled at 95°C for 10 min. The eluted proteins were separated on 9% SDS-polyacrylamide gel and detected through Western blot analysis with anti-p-FoxO3a (1:200 dilution).

### ELISA assay

The levels of myostatin, activin A, TNF-α, IL-6 and IL-1β were determined using respective ELISA kits from R&D Systems, Inc. (MN, USA).

### Histological examination

Intestinal segments were fixed with 10% formaldehyde and embedded in paraffin followed by hematoxylin and eosin staining to evaluate the pathological changes. Intestinal injury was scored according to the histopathological grading system [35] in accordance with the following principle: 0. normal histological findings; 1. mucosa: villus blunting, loss of crypt architecture, sparse inflammatory cell infiltration, vacuolization and edema/normal muscular layer; 2. mucosa: villus blunting with fattened and vacuolated cells, crypt necrosis, intense inflammatory cell infiltration, vacuolization and edema/normal muscular layer; and 3. mucosa: villus blunting with fattened and vacuolated cells, crypt necrosis, intense inflammatory cell infiltration, vacuolization and edema/muscular edema. To determine the score of tissue injury, histological images were examined by a trained pathologist who was initially blinded to these groups.

### Intestinal digestion enzyme activity assay

Intestine extracts were prepared in 0.9% NaCl supplemented with a proteinase inhibitor, and then the major digestive enzyme activity of the intestines, including LAP, LIP, and AMYL, was measured. The biochemical tests were conducted using a Fuji DRI-CHEM 3030 Analyzer (Fuji Photo Film Co. Ltd., Tokyo, Japan).

### Statistical analysis

The experimental data are expressed as the mean ± standard error of the mean. One-way analysis of variance with a post hoc Bonferroni test was used for statistical analysis. Results were considered significantly different at a *P* value < 0.05.
